# Surface Plasmon’s Dispersion Properties of Porous Gold Films

**DOI:** 10.1186/s11671-016-1327-7

**Published:** 2016-02-29

**Authors:** M. O. Stetsenko, L. S. Maksimenko, S. P. Rudenko, I. M. Krishchenko, A. A. Korchovyi, S. B. Kryvyi, E. B. Kaganovich, B. K. Serdega

**Affiliations:** V. Lashkaryov Institute of Semiconductor Physics, National Academy of Sciences of Ukraine, 45, Pr. Nauky, Kyiv, 03028 Ukraine

**Keywords:** Gold nanoparticles, Porous films, Surface plasmons, Localized and propagating modes, Dispersion properties, Spatial dispersion, PLD, MPS

## Abstract

Nanostructure porous films with arrays of gold nanoparticles (Au NPs) have been produced by pulsed laser deposition. Dispersion properties of surface plasmons have been studied by the modulation-polarization spectroscopy technique. The dispersion relations for radiative modes and two types of non-radiative modes of localized and propagating surface plasmons were obtained. The branches of propagating modes were characterized by negative group velocity caused by spatial dispersion of dielectric function. The propagating modes are caused by dipole-dipole interactions between adjacent Au NPs. The frequencies and relaxation parameters of surface plasmon resonances and the plasma frequencies for Αu NPs were obtained. The relation between the surface plasmon’s properties and formation conditions of films with arrays of Αu NPs is discussed.

## Background

The interaction between electromagnetic radiation and electrons in metal nanostructures is accompanied by surface plasmon resonance (SPR) manifesting itself. There are surface plasmon-polariton (SPP) resonances with the propagating wave on an infinite flat metal-dielectric surface and a localized surface plasmon (LSP) resonance on separated non-interacting metal nanoparticles and between metal nanoparticles due to electrodynamic/dipole-dipole interactions [1–5]. Dispersion characteristics *ω*(*k*) of surface plasmons contain information about optical properties and nature of surface plasmon excitations and structure features of investigated films. It is known that one branch of the dispersion characteristics *ω*(*k*) determines the SPP resonance for homogeneous gold nanostructure. The dependencies of *ω*(*k*) split into several branches for nanocomposites and nanostructures with arrays of gold nanoparticles (Au NPs) due to dipole-dipole interactions between nanoparticles. Both the resonances and the dispersion characteristics of surface plasmons are characterized by considerable variety [6–19].

A particular interest for investigation of dipole-dipole interactions between Au NPs is nanostructure porous films [20–25]. One of the most effective formation methods of film nanostructures of noble metals is pulsed laser deposition (PLD). Detailed analysis of the dispersion relations of surface plasmons has not been investigated in our earlier works [26–28], which studied the transmission spectra, structural properties, and surface morphology for porous metal films (por-Au(Ag)) by atomic force microscopy (AFM), scanning electron microscopy (SEM), and X-ray reflectometry (XRR) methods. At a recent time, an effective diagnostic method for the studying of plasmonic nanostructures is the modulation-polarization spectroscopy (MPS) technique. In the works [29–32], the SPR features have been studied by MPS for clustered Au films and nanocomposite films with Au NPs in various matrices.

The aim of this work is to study the dispersion properties of surface plasmons using the MPS technique for porous gold films with randomly distributed Au NP arrays obtained by PLD.

## Methods

### Sample Preparation

Porous gold films (por-Au) have been produced by the pulsed laser deposition method with a YAG:Nd^3+^ laser (a wavelength of 1.06 μm, laser fluence of 15 j/cm^2^, pulse duration of 10 ns, repetition frequency of 25 Hz) in an argon atmosphere with a variable pressure *P*_Ar_ and pulse number *N*. For *N* = 3 · 10^3^ and 15 · 10^3^, obtained samples 1 and 1′ correspond to *Р*_Аr_ = 20 Pa and samples 2 and 2′ to *Р*_Аr_ *=* 70 Pa, respectively (see Table 1). The deposition was performed from the direct flux of Au NPs of erosion torch on glass substrate, which is placed on the distance of 30 mm along the normal to the target plain.

**Table 1:** Various parameters for porous gold films

	Sample no.	1	2	1′		2′	
PLD	*P*_Ar_, Pa	20	70	20		70	
	*N* ∙ 10^3^	3	3	15		15	
ΑFM	*D*, nm	10.8 (4.9–22.7)	6.9 (3.6–12.2)	25.5 (3–56.4)		16.3 (3.2–31.7)	
	*R*, nm	40 (25.3–71.84)	9.7 (6–16.7)	19 (9.1–29.9)		11.9 (4.4–18.9)	
	Average neighbor distance, nm	40	9.7	19		11.9	
	*q*	0.3	0.23	1		1	
XRR	Density NP ∙ 10^3^/μm^2^	3	5.5	1.7		4.3	
	Porosity, %			30.1		36.6	
	*d*, nm	–	–	10.6		6	
*T*(*λ*)	*λ*_min_, nm	680	740	620		560	
*θ*_*ρ*=0_(*λ*)	*λ*_р_, nm (*ω*_р_ ⋅ 10^15^, Hz)	552 (3.415)	590 (3.195)	508 (3.71)		515 (3.66)	
*ρ*(*λ*) *θ* = 45°–60° (70)°	*ω*_sp1_ ∙ 10^15^, Hz	4.25–(4.20)	4.23–(4.32)	4.34–4.33		4.3–4.37	
	*γ*_sp1_ ∙ 10^15^, s^−1^	0.57–(0.63)	0.52–(1.11)	1–1.08		0.96–1.03	
	*ω*_sp2,3_ ∙ 10^15^, Hz	3.29–(3.18)	3.14–(2.76)	3.1–2.04	2.08–1.85	3.34–2.4	2.4–1.97
	*γ*_sp2,3_ ∙ 10^15^, s^−1^	1.35–(1.36)	1.62–(1.29)	1.4–1.14	1.04–0.36	1.08–0.98	1.27–0.4

### Methods

Surface morphology of the deposited films was studied by atomic force microscopy using microscope Nanoscope IIIa (Digital Instruments) in a periodical contact regime with probes at a nominal tip radii of 10 nm.

In order to determine the density and the thickness of por-Au films, the X-ray reflectometry investigations were performed using a high-resolution Panalytical X’Pert PRO MRD system with Cu_Kα1_ radiation and a standard four-bounce Ge (220) monochromator. The incident X-ray beam was collimated with a 0.1-mm gap.

Transmission spectra of the films were measured using a spectrophotometer SF-26 within the wavelength range *λ* = 0.36–1 μm.

Angular and spectral polarization characteristics of por-Au films were measured in Kretschmann geometry by the modulation-polarization spectroscopy technique in the wavelength range *λ* = 0.4–1 μm. The setup scheme is described in detail in [29]. The MPS technique is based on the modulation of polarization of electromagnetic radiation, when the *s*- and *p*-polarizations are alternately transformed at a constant intensity, frequency, phase, and wave vector. The value of an experimental registered signal on a modulation frequency (*f* = 60 kHz) is the polarization difference *ρ*(*λ*,*θ*) = *R*_s_^2^ *– R*_p_^2^, which is a magnitude of difference between the intensities of the internal reflection coefficients of *s*- and *p*-polarized radiation. According to the conventional terminology in polarimetry [33], the parameter *ρ* is a *Q*-component of the Stokes vector, which is a part of the reflected radiation in our case from the system of quartz half-cylinder—por-Au film—air. The parameter *ρ* is more informative for detection of small changes of signals with high sensitivity to morphology features of the nanostructures due to an simultaneous measuring of *R*_s_^2^ and *R*_p_^2^ coefficients under interaction between the radiation and sample. The refractive index of the quartz half-cylinder *n* = 1.456 determines the value of the critical angle of the total internal reflection as *θ*_cr_ = 43.6°. When the reflection coefficients of *s*- and *p*-polarized radiation have equal amplitude values, i.e., *R*_s_^2^(*θ*) *= R*_p_^2^(*θ*), and the magnitude of polarization difference *ρ*(*θ*) equals zero, the light reflection occurs regardless of polarization state at the angle of isotropic reflection *θρ =* 0 [32]. Spectral dependencies of the angle of isotropic reflection *θρ =* 0(*λ*) were measured to obtain values of the plasma frequency oscillations of the electrons in Au NPs.

## Results and Discussion

### AFM and XRR Studies

**Fig. 1:**
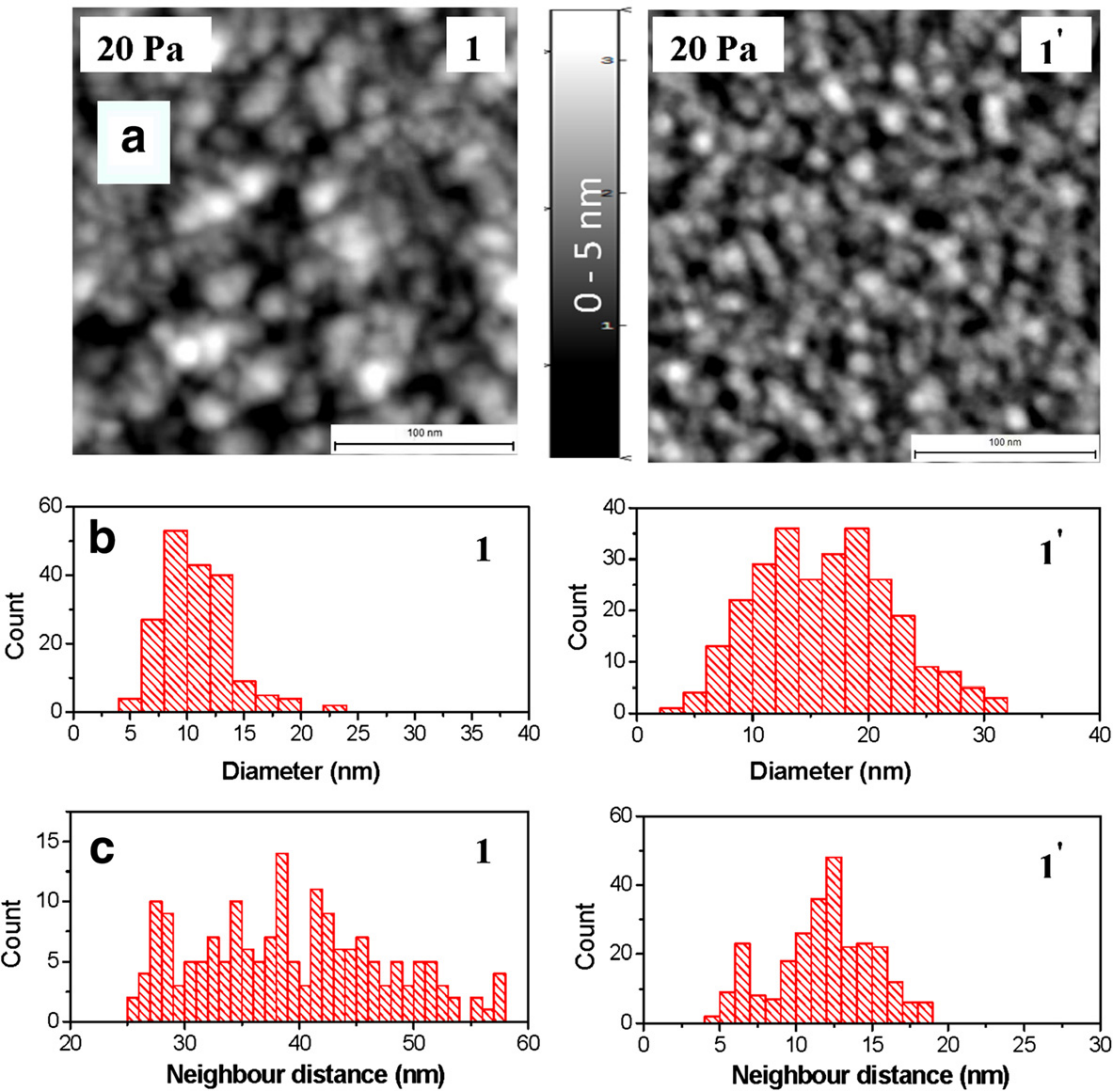
AFM images (**a**), histograms of distribution of Au NP size (**b**), and neighbor distance between Au NPs (**c**) for por-Au films at *P*_Ar_ = 20 Pa (samples *1* and *1′*)

Figure 1a shows AFM images of samples 1 and 1′ obtained at *P*_Ar_ = 20 Pa for *N* = 3 · 10^3^ and *N* = 15 · 10^3^, respectively. The corresponding histograms of distribution of Au NP size and neighbor distance between Au NPs are presented in Fig. 1b, c respectively. These two samples of relatively broad size distributions and interparticle distances are characterized by an irregular nanostructure with randomly distributed Au NPs of spherical and spheroid shapes. In principle, the samples 2 and 2′ at *P*_Ar_ = 70 Pa have a similar morphology surface. A distinctive feature for samples 1′ and 2′ at *N* = 15 · 10^3^ is a high density of conjunction and aggregation of Au NPs. For samples 1 and 2 at *N* = 3 · 10^3^, with increasing argon pressure from *P*_Ar_ = 20 to 70 Pa, the Au NP heights (*h*) are decreasing from 5–6 to 4 nm; an average diameter (*D*)—from 10.8 to 6.9 nm; size particle dispersion—from 5–22 to 3.6–12 nm; interparticle distance—from 25–72 to 6–17 nm; coverage area of Au NPs (*q*)—from 30 to 23 % (see Table 1). The interparticle distances of Au NPs at *P*_Ar_ = 20 Pa exceed their sizes more than 2.5 times and lessen at *P*_Ar_ = 70 Pa. Consequently, both samples 1 and 2 are characterized by an island 2D structure, on the one hand, with isolated non-interacting Au NPs at *P*_Ar_ = 20 Pa, and on the other hand, with dipole’s field interactions between adjacent Au NPs at *P*_Ar_ = 70 Pa.

The X-ray reflectometry measurements were performed only for samples 1′ and 2′ at *N* = 15 · 10^3^ that was not done for samples 1 and 2 due to the island structure of these films. According to the XRR results, with increasing argon pressure, the density of samples 1′ and 2′ is decreased from 13.5 to 12.24 g/cm^2^ in comparison with the density of bulk gold of 19.32 g/cm^2^. Consequently, if porosity of the films increases from 30.1 to 36.6 %, the film thickness is reduced from 10.6 to 6 nm. Such dependencies of structural and optical properties of por-Au films on their formation conditions are defined by the formation processes at the PLD in the presence of argon in a torch, out of the torch, and on the substrate. This formation process depends on the basic deposition parameters such as argon pressure *P*_Ar_ and pulse number *N*.

As shown in [20, 25], the morphology features of porous films with Au NP arrays depend on formation conditions at both the PLD and the high-frequency sputtering methods. There are many small nanoparticles at low argon pressure *P*_Ar_ in a torch and a few large nanoparticles at high *P*_Ar_. With increasing of *P*_Ar_, a torch is compressed, the interaction between the Au atoms is increased in the torch, and also the distance between the torch edge and the substrate is increased. Outside the torch, the interaction between Au NPs and Ar atoms is weak at low *P*_Ar_, and at a high *P*_Ar_, both the mean free path and the kinetic energy are increased. The deposition of the film onto the substrate occurs from the flow of high-energy Au NPs of high density. The rate of NP formation is large, which leads to the growth in size of the NPs. On the other hand, at high *P*_Ar_, the interaction between Au NPs and Ar atoms is strong. Both the mean free path and the kinetic energy. The deposition of the film occurs from the flow of low-energy Au NPs of low density. Both the number and the average size of Au NPs on the substrate are decreased. A significant amount of larger low-energy Au NPs take part in the reverse transfer onto the substrate, which is placed on the distance along the normal to the target plain.

### UV-Visible Spectroscopy

**Fig. 2:**
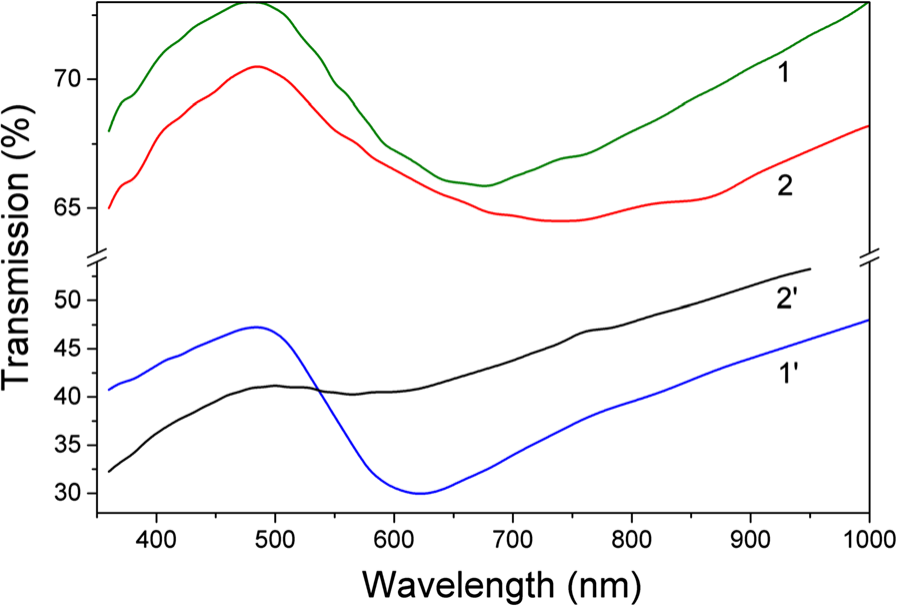
Transmission spectra of por-Au films at different argon pressures *P*_Ar_ (20 and 70 Pa) and pulse numbers *N* (3 and 15 · 10^3^) for samples *1*, *1′*, *2*, and *2′*, respectively

Figure 2 presents the transmission spectra *T*(*λ*) for samples 1, 1′, 2, and 2′. All curves of *T*(*λ*) exhibit the typical extrema of localized surface plasmon resonances. The absorption bands of surface plasmons are broad with extrema positions within the range of 560–740 nm, which are shifted to long-wavelength range compared to the Frohlich dipole mode (*λ* ≈ 520 nm) for small Au NPs. When *N* increases from 3 · 10^3^ to 15 · 10^3^, the transmission coefficient of radiation is monotonically decreased according to the order of samples 1, 2, 1′, and 2′. For samples 1′ and 2′, typically with increasing *P*_Ar_ from 20 to 70 Pa, the wavelength value in the LSP resonance is decreased from 620 to 560 nm. As for samples 1 and 2 with similarly increasing *Р*_Аr_, these values are increased from 680 to 740 nm.

### MPS Study

#### Spectroscopy of Polarization Difference

**Fig. 3:**
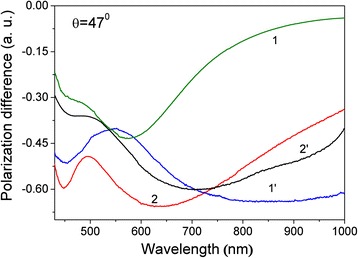
Spectral dependences of the polarization difference *ρ*(*λ*) for samples *1*, *1′*, *2*, and *2′* of por-Au films at an incident angle of *θ* = 47*°*

Figure 3 shows the spectral characteristics of polarization difference *ρ*(*λ*) at an incident angle of *θ =* 47*° > θ*_cr_. For all samples 1, 2, 1′, and 2′, the amplitudes of *ρ*(*λ*) have negative values due to increasing of the absorption of *s-*polarized radiation, as compared with the *p-*polarized one, i.e., *R*_s_^2^(*λ*) < *R*_p_^2^(*λ*). This is typical for island nanostructures and nanocomposite films with Au NP arrays. The bands of spectral characteristics of *ρ*(*λ*) are broadened with extrema, which are explained by different types of the LSP resonance. On the one hand, extremum in a short-wavelength range is caused by resonant excitations of surface plasmons on isolated non-interacting Au NPs. On the other hand, extremum in a long-wavelength range is caused by resonant excitations of surface plasmons between NPs due to electrodynamic/dipole-dipole interactions of adjacent NPs. As a result, the surface plasmon oscillations in a single Au NP induces the surface plasmon oscillations in adjacent Au NPs when the phase synchronism condition is satisfied and both frequency (*ω*) and wave vector (*k*) of light excitation match those of the surface plasmon’s frequency and wave vector. Hence, the SPR manifests itself. Moreover, both spectra of transmission *T*(*λ*) and polarization difference *ρ*(*λ*) have a correlation in the extrema position and their redshift relative to each other.

**Fig. 4:**
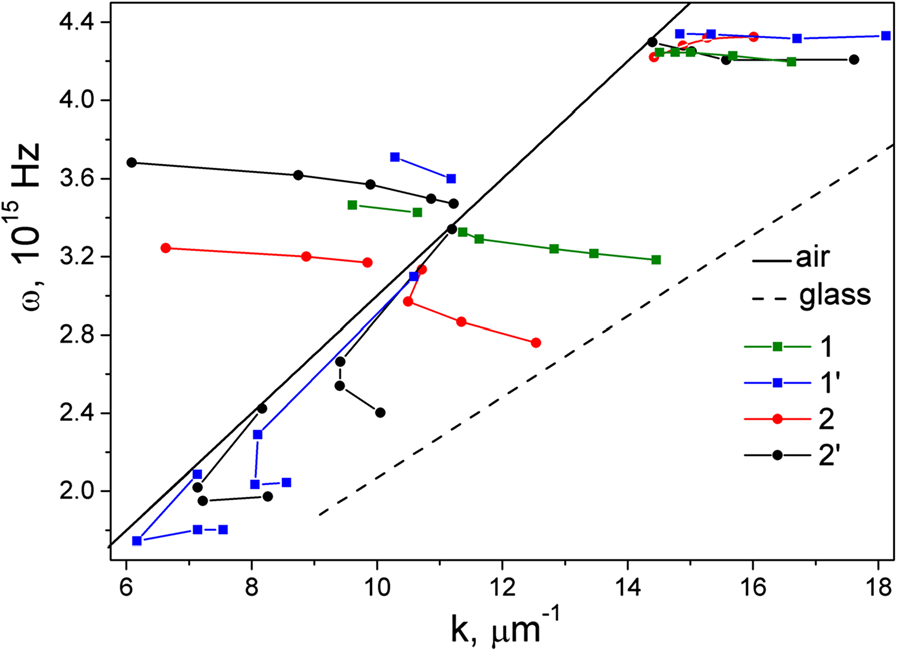
Dispersion characteristics *ω*(*k*) of surface plasmons for samples *1* and *1′* (*squares*) and *2* and *2′* (*circles*) of por-Au films

To reveal the underlying features and clarify the extremum positions and intensities, all spectra of *ρ*(*λ*) in a wide range of incident angles *θ* were converted to appropriate frequency dependencies *ρ*(*ω*) and then decomposed into elementary components that are described by Gaussian functions. As a result, the main resonance parameters of the samples as fundamental frequencies and relaxation times have been obtained and summarized in Table 1. With the help of the frequency data, the dispersion characteristics *ω*(*k*) of surface plasmons were obtained for samples 1, 2, 1′, and 2′, which are shown in Fig. 4. A distinctive feature of the dispersion relations is that the dispersion is much larger within the range of incident angles of *θ* > *θ*_cr_, which corresponds to the surface plasmon’s excitations of non-radiative modes as compared with radiative modes at *θ* < *θ*_cr_. The dispersion branches of radiative modes (to the left of the light straight line) are explained by the surface plasmon’s excitations on isolated non-interacting Au NPs. Note that with increasing of argon pressure *P*_Ar_, the frequency magnitude of radiative modes is decreased for samples 1 and 2 at *N* = 3 · 10^3^ and samples 1′ and 2′ at *N* = 15 · 10^3^. This dispersion feature of *ω*(*k*) for radiative modes is caused by a reduction in size of Au NPs.

Two groups of dispersion branches are shown for non-radiative modes (to the right of the light straight line) where the phase synchronism condition is necessary for the surface plasmon’s excitations. High-frequency branches *ω*_1_ are independent on the incident angle *θ*, which indicates localization of surface plasmons. Their frequency ranges are narrow (4.2 – 4.37) · 10^15^ Hz because of weak dependencies of ω(k) on the incedent angels *θ* for different por-Au films. In this case, the surface electromagnetic wave does not develop, and its propagation is similar to a standing wave. The low-frequency branches *ω*_2_ and *ω*_3_ correspond to the propagation of the surface plasmon’s excitations between Au NPs due to dipole fields’ interactions with adjacent NPs (the influence of neighboring Au NPs is dominant). These surface plasmon’s excitations occur in broadened frequency ranges of (1.85–3.34) · 10^15^ Hz and strongly depend on the incident angles. One can see the correlation in the arrangement of low-frequency branches *ω*(*k*) with the corresponding order of the transmission spectra for samples 1, 2, 2′, and 1′.

Different surface morphology features are exhibited in dispersion dependencies of surface plasmons, in particular for 2D and 3D nanostructures the surface features are demonstrated in the curves 1,2 and 2´,1´, respectively. On the one hand, the surface morphology of 2D nanostructures at *P*_Ar_ = 20 Pa is characterized by bigger size and interparticle distances of Au NPs and low coverage density of NPs due to the presence of isolated Au NPs. On the other hand, with increasing of *P*_Ar_ = 70 Pa, the interactions between Au NPs are added. Note that the surface morphology of 3D nanostructures is characterized by the presence of Au NP aggregates, high-packing density of Au NPs, variety of incident angles, and wide distributions of size, shape, and interparticle distance. Their low-frequency modes of surface plasmons are split into two branches with a wide range of relaxation times. In addition, an important feature of low-frequency branches of samples 2, 2′, and 1′ is a negative group velocity in the ratio of *dω*(*k*)/*dk*.

It is known from [34, 35] that negative group velocity can be realized for the media with spatial dispersion of the dielectric properties, which is caused by dependence of the dielectric tensor not only on the frequency but also on the wave vector *ε*_*ij*_(*ω*, *k*). Consequently, the non-locality of the dielectric response to electromagnetic excitation takes place [36]. Similar negative dependencies of *ω*(*k*) were observed in works [4, 37] for films with chains of Au NP arrays. In our case, for samples 1′ and 2′ with 3D structures of por-Au films and close-packed arrays of plasmonic nanoparticles, the short-range order occurs due to strong electrodynamic interparticle interactions. Therefore, for por-Au films and the chains of Au NP arrays, the negative group velocity of surface plasmons can appear.

#### Spectroscopy of Angle of Isotropic Reflection

**Fig. 5:**
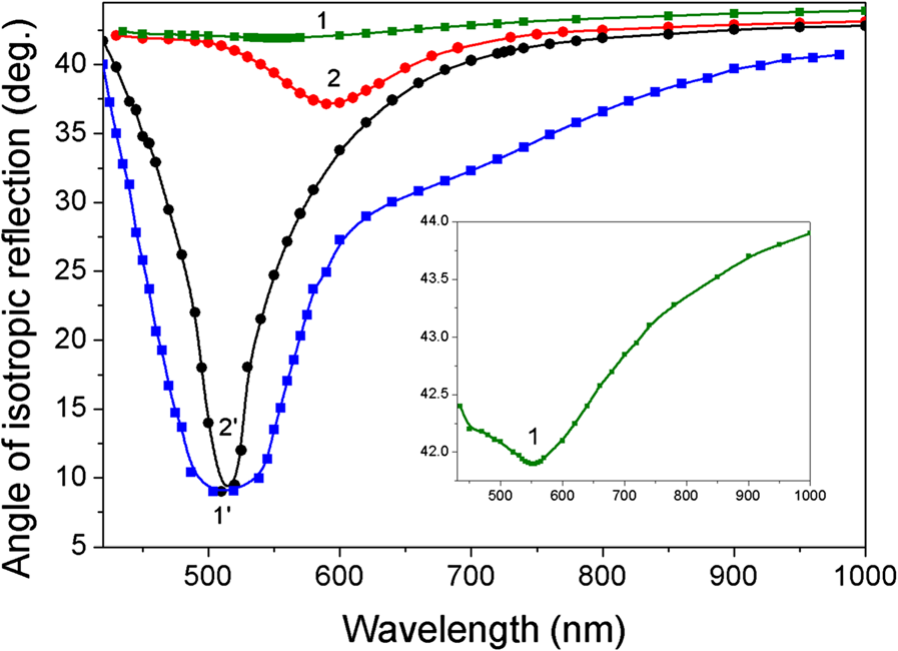
Spectral dependences of the isotropic reflection angles *θρ = 0*(*λ*) for samples *1* and *1′* (*squares*) and *2* and *2′* (*circle*s) of por-Au films

To understand the resonant-optical properties of por-Au films, the spectral characteristics of the angle of isotropic reflection *θρ = 0*(*λ*) are shown in Fig. 5 in a radiative range of *θ < θ*_cr_ for samples 1, 1′, 2, and 2′. The isotropic reflection of radiation (*R*_s_^2^ *= R*_p_^2^) occurs in the following cases: the first is a normal transmission/reflection of non-polarized radiation; the second is an attenuated internal reflection, when *R*_s_^2^ *< R*_p_^2^. Each of these coefficients does not necessarily need to be zero. The equality of their magnitude is important. The last case in the present work was realized by the MPS method. All curves of *θρ = 0*(*λ*) exhibit resonance character with extrema, which are defined by the plasmons’ oscillations that are correlated to the natural oscillations of the conduction electrons (plasma frequency *ω*_р_) for Au NPs [32]. Determination of the plasma frequency of nanomaterials in different environments is an important issue for plasmonics. The bands of spectral characteristics of *θρ = 0*(*λ*) are broadened with a decreasing interparticle distance of Au NPs due to the increasing of interaction of adjacent Au NPs. The plasma oscillations of electrons in Au NPs are radiative modes. Hence, its position on dispersion branches of *ω*(*k*) lies also in the radiative region in Fig. 4. Obtained values of *ω*_р_ are equal to 3.415, 3.195, 3.71, and 3.64 (⋅10^15^) Hz for samples 1, 2, 1′, and 2′, respectively. Note that these *ω*_р_ values are less than the corresponding bulk material of gold of 1.37 ⋅ 10^16^ Hz. Moreover, the values of *ω*_р_ are reduced for both 2D and 3D nanostructures and also reduced relative to each other due to decreasing of Au NP size in por-Au films. It is known that plasma frequency depends on electron concentration in each separated metal film [1, 3]. Moreover, if the size of NPs is comparable to the electron mean free path (in the size range of 1–10 nm), then the electrons are able to spill out of the Au NPs. This quantum size effect can lead to changing of plasma frequency value *ω*_р_ with the decreasing size of Au NPs due to the finite potential barrier at the surface assuming a sharp interface between a particle and the surrounding medium [38].

One can see that the extremum of *θρ = 0*(*λ*) has a redshift with decreasing plasma frequency *ω*_р_ for samples 1 and 2 with 2D nanostructures. Consequently, the resonant character of dependencies can be explained by a modified Mie theory based on time-dependent local density approximation (TDLDA), i.e., taking into account the effect of an electron’s spill out of the nanoparticles while decreasing their size. On the other hand, for 2D nanostructures, a wide plasmon’s absorption bands were observed in the transmission spectra *T*(*λ*). Full width at half maximum (FWHM) values for samples 1 and 2 are increased from 300 to 395 nm. A band width of absorption band *Г* is determined by the relaxation time of the electron oscillations *τ* in the relation of $\gamma \sim {\tau}^{-1}\sim \frac{A{V}_F}{L_{\mathrm{eff}}}$, where *V*_*F*_ is a Fermi velocity, *L*_eff_ is an effective electron mean free path, and *A* is a non-dimensional parameter, which is defined by features of electrons scattering on the nanoparticle surface. The relaxation time is reduced, and the plasmon’s absorption band is broadened. Hence, broadening of *T*(*λ*) bands with the decreasing of Au NP size correlates with an increasing of parameter *A* due to the surface elastic scattering of the electrons. The nature of resonant-optical properties of por-Au films with 3D nanostructures differs from those with 2D nanostructures. As has been shown for samples 1′ and 2′ with a 3D nanostructure, the blueshifts of *θρ = 0*(*λ*) resonances with the decreasing of the average size of Au NPs from 25.5 to 16.3 nm were observed in contrast to those redshifts for samples 1 and 2 with 2D nanostructures. As shown in [39], similar shifting of plasmon resonances in both red- and blue-wavelength directions can be caused by the changing of the Au NP shape from spherical to spheroidal. Moreover, with a decreasing of Au NP size, the plasmon’s absorption band does not broaden but narrows from 225 to 85 nm. There is no correlation in changing the frequency extrema between LSP resonance and plasma frequency of the electrons for appropriate por-Au films. The nature of their resonant-optical properties is determined obviously by binding and hybridization of the dipole’s fields of Au NPs in the closed-packed arrays [40].

## Conclusions

Both 2D and 3D nanostructures with randomly distributed Au NP arrays of porous gold films have been performed by the PLD method from the direct high-energy flow of erosion-torch particles in an argon atmosphere by varying the argon pressure and pulse number. For the first time, the dispersion properties of surface plasmons for por-Au films have been studied by the MPS technique. Transmission spectra of the films were typical for localized surface plasmon resonances. Different types of LSP resonances were obtained with the help of the spectral characteristics of the polarization difference *ρ*(*λ*) and the isotropic reflection angles *θρ = 0*(*λ*). The first type is explained by the surface plasmon’s excitation on isolated non-interacting Au NPs (dipole and multipole modes), and the second is between adjacent Au NPs caused by dipole-dipole interactions. The dispersion relations for radiative modes and two types of non-radiative modes of localized and propagating surface plasmons were demonstrated. The branches of propagating modes were characterized by negative group velocity caused by spatial dispersion of dielectric function. The frequencies and relaxation parameters of surface plasmon resonances and the plasma frequencies for Αu NPs were obtained and discussed in correlation with the size, shape, and morphology structures of por-Au films. It was demonstrated that the plasma frequencies *ω*_р_ are reduced for both 2D and 3D nanostructures and also reduced relative to each other due to the decreasing of Au NP size in por-Au films.

## References

[1] Kreibig U, Volmer M (1995). Optical properties of metals clusters.

[2] Dmitruk NL, Goncharenko AV, Venger EF (2009). Optics of small particles and composite media.

[3] Maier SA (2007). Plasmonic fundamentals and application.

[4] Dmitruk NL, Malinich SZ (2014). Surface plasmon resonances and their manifestation in the optical properties of nanostructures of noble metals. Ukrainian Journal of Physics Reviews.

[5] Le Ru E, Etchegoin P (2009). Principles of surface enhanced Raman spectroscopy and related plasmonic effects.

[6] Diaz-Egea C, Abargues R, Martinez-Pastor JP, Sigle W, van AkenP A, Molina SI (2015). High spatial resolution mapping of individual and collective localized surface plasmon modes of silver nanoparticle aggregates: correlation to optical measurements. Nanoscale Res Lett.

[7] Su K-H, Wei Q-H, Zhang X, Mock JJ, Smith DR, Schultz S (2003). Interparticle coupling effects on plasmon resonances of nanogold particles. Nanoletters.

[8] Halas NJ, Lai S, Chang W-S, Link S, Nordländer P (2011). Plasmons in strongly coupling metallic nanostructures. Chem Rev.

[9] Kundu S (2012). Interparticle coupling of plasmon fields due to reorganization of Au nanoparticles in Langmuir-Blodgett film. J Appl Phys.

[10] Choi B, Lee H-Н, Jin S, Chun S, Kim S-H (2007). Characterization of the optical properties of silver nanoparticle films. Nanotechnology.

[11] Zamkovets AD, Kachan SM, Ponyavina AN (2008). Concentration-related enhancement of the sensitivity of surface plasmon resonance of metallic nanoparticles to the characteristics of a dielectric environment. J Appl Spectrosc.

[12] Quinten М (2001). Local fields close to the surface of nanoparticles and aggregates of nanoparticles. Appl Phys B Lasers Opt.

[13] Khurgin JB, Sun G (2009). Impact of disorder on surface plasmons in two-dimensional arrays of metal nanoparticles. Appl Phys Lett.

[14] Nishijima Y, Rosa L, Juodkazic S (2012). Surface plasmon resonances in periodic and random patterns of gold nano-disks for broadband light harvesting. OpticsExpress.

[15] Khlebtsov NG (2008). Optics and biophotonics of nanoparticles with a plasmon resonance. Quantum electronics.

[16] Zou S, Janel N, Schatz G (2004). Silver nanoparticle array structures that produce remarkably narrow plasmon line shapes. The Journal of Chemical Physics.

[17] Haynes CL, McFarland AD, Zhao LL, Van Duyne RP, Schatz GC (2003). Nanoparticle optics: the importance of radiative dipole coupling in two-dimensional nanoparticle arrays. J Phys Chem B.

[18] Lampreecht B, Schider G, Lechner RT, Ditlbacher H, Krenn JR, Leitner A, Aussenegg FR (2000). Metal nanoparticle gratings: influence of dipolar particle interaction on the plasmon resonance. Phys Rev Lett.

[19] Atay T, Song J-H, Nurmikko AV (2004). Strongly interacting plasmon nanoparticle pairs: from dipole-dipole interaction to conductively coupled regime. Nanoletters.

[20] Kabashin AV, Meunier M (2006). Recent advances in laser processing of materials.

[21] Pan Z, Zabalin A, Veda A, Guo M, Groza M, Burger A, Mu R, Morgan SH (2005). Surface-enhanced Raman spectroscopy using silver-coated porous glass-ceramic substrates. Appl Spectrosc.

[22] Yu F, Ahl S, Caminade AM, Majoral JP, Knoll W, Erlebacher J (2006). Simultaneous excitation of propagating and localized surface plasmon resonance in nanoporous gold membranes. Anal Chem.

[23] Qian LH, Yan XQ, Fujita T (2007). Surface enhanced Raman scattering of nanoporous gold: smaller pore sizes stronger enhancements. Appl Phys Lett.

[24] Schilling J, Sardana N, Heyroth F (2012). Propagating surface plasmons on nanoporous gold. Metamaterials 2012: The VI International Congress on Advanced Electromagnetic Materials in Microwaves and Optics.

[25] Agarwal NR, Neri F, Trusso S, Lucotti A, Ossi PM (2012). Au nanoparticle arrays produced by pulsed laser deposition for surface enhanced Raman spectroscopy. Appl Surf Sci.

[26] Kladko VP, Gudymenko OY, Kriviy SB, Litvin PM, Kaganovich EB, Krishchenko IM, Manoilov EG (2014). Reflectometry study of nanoporous films with arrays of gold nanoparticles. Ukrainian Journal of Physics.

[27] Strelchuk VV, Kolomys ОF, Kaganovich ЕВ, Krishchenko IM, Golichenko ВО, Boyko МI, Kravchenko SО, Kruglenko IV, Lytvyn ОS, Manoilov EG (2015). Optical characterization of SERS substrates based on porous Au films prepared by pulsed laser deposition. J Nanomater.

[28] Kaganovich EB, Krishchenko IM, Manoilov EG, Maslak-Gudyma NP, Kremenitskiy VV (2012). Structure and optical properties of gold and silver porous films obtained by pulsed laser deposition in vacuum. Nanosystems, nanomaterials, nanotechnologes.

[29] Berezhinsky LJ, Maksimenko LS, Matyash IE, Rudenko SP, Serdega BK (2008). Polarization modulation spectroscopy of surface plasmon resonance. Opt Spectrosc.

[30] Berezhinskiі LI, Litvin OS, Maksimenko LS, Matyash IE, Rudenko SP, Serdega BK (2009). Size effects in the internal reflection in gold cluster films in polarization modulation experiments. Opt Spectrosc.

[31] Kaganovich EB, Kravchenko SA, Maksimenko LS, Manoilov EG, Matyash IE, Mishchuk ON, Rudenko SP, Serdega BK (2011). Polarization properties of porous gold and silver films. Opt Spectrosc.

[32] Maksimenko LS, Matyash IE, Mischuk OM, Stetsenko MO, Serdega BK (2015). Diagnostic of surface plasmons resonances in nanosized gold films by modulation polarization spectroscopy. Plasmonics.

[33] Gerrard A, Burch JM (1975). Introduction to matrix methods in optics.

[34] Agranovich VM, Gartstein YN (2006). Spatial dispersion and negative refraction of light. Physics-Uspekhi.

[35] Fedyanin DY, Arsenin AV, Leiman VG, Gladun AD (2009). Surface plasmon-polaritons with negative and zero group velocities propagating in thin metal films. Quantum Electronics.

[36] Ruppin R (2001). Surface polaritons of a left-handed material slab. J Phys Condens Matter.

[37] Yang T, Crozier KB (2008). Dispersion and extinction of surface plasmons in an array of gold nanoparticle chains: influence of the air/glass interface. OpticsExpress.

[38] Monreal RC, Antosiewicz TJ, Apell SP (2013). Competition between surface screening and size quantization for surface plasmons in nanoparticles. New J Phys.

[39] Gupta G, Tanaka D, Ito Y, Shibata D, Shimojo M, Furuya K, Mitsui K, Kajikawa K (2009). Absorption spectroscopy of gold nanoisland films: optical and structural characterization. Nanotechnology.

[40] Song J-H, Hong S-Y, Kim Y-G, Lee K-W, Kim Y-Y (2007). Observation of plasmon hybridization in gold nanoparticle pairs. Journal of the Korean Physical Society.

